# Olive- and Coconut-Oil-Enriched Diets Decreased Secondary Bile Acids and Regulated Metabolic and Transcriptomic Markers of Brain Injury in the Frontal Cortexes of NAFLD Pigs

**DOI:** 10.3390/brainsci12091193

**Published:** 2022-09-04

**Authors:** Magdalena A. Maj, Tanvi R. Gehani, Chad Immoos, Mikaelah S. Medrano, Rob K. Fanter, Christine R. Strand, Hunter Glanz, Brian D. Piccolo, Mohammed K. Abo-Ismail, Michael R. La Frano, Rodrigo Manjarín

**Affiliations:** 1 Department of Biological Sciences, California Polytechnic State University, 1 Grand Ave., San Luis Obispo, CA 93407, USA; 2 Center for Applications in Biotechnology, California Polytechnic State University, 1 Grand Ave., San Luis Obispo, CA 93407, USA; 3 Department of Biomedical Engineering, California Polytechnic State University, 1 Grand Ave., San Luis Obispo, CA 93407, USA; 4 Department of Chemistry and Biochemistry, California Polytechnic State University, 1 Grand Ave., San Luis Obispo, CA 93407, USA; 5 College of Agriculture, Food and Environmental Sciences, California Polytechnic State University, 1 Grand Ave., San Luis Obispo, CA 93407, USA; 6 Cal Poly Metabolomics Service Center, California Polytechnic State University, 1 Grand Ave., San Luis Obispo, CA 93407, USA; 7 Department of Statistics, California Polytechnic State University, 1 Grand Ave., San Luis Obispo, CA 93407, USA; 8 USDA-ARS Arkansas Children’s Nutrition Center, 1120 Marshall St. SLOT 512-20B, Little Rock, AR 72202, USA; 9 Department of Pediatrics, University of Arkansas for Medical Sciences, 4301 W Markham St., Little Rock, AR 72205, USA; 10 Department of Animal Science, California Polytechnic State University, 1 Grand Ave., San Luis Obispo, CA 93407, USA; 11 Department of Food Science and Nutrition, California Polytechnic State University, 1 Grand Ave., San Luis Obispo, CA 93407, USA

**Keywords:** transcriptomics, metabolomics, Iberian pigs, brain, pediatric model, neurodegeneration

## Abstract

The objective of this study was to investigate the effect of dietary fatty acid (FA) saturation and carbon chain length on brain bile acid (BA) metabolism and neuronal number in a pig model of pediatric NAFLD. Thirty 20-day-old Iberian pigs, pair-housed in pens, were randomly assigned to receive one of three hypercaloric diets for 10 weeks: (1) lard-enriched (LAR; *n* = 5 pens), (2) olive-oil-enriched (OLI, *n* = 5), and (3) coconut-oil-enriched (COC; *n* = 5). Pig behavior and activity were analyzed throughout the study. All animals were euthanized on week 10 and frontal cortex (FC) samples were collected for immunohistochemistry, metabolomic, and transcriptomic analyses. Data were analyzed by multivariate and univariate statistics. No differences were observed in relative brain weight, neuronal number, or cognitive functioning between diets. Pig activity and FC levels of neuroprotective secondary BAs and betaine decreased in the COC and OLI groups compared with LAR, and paralleled the severity of NAFLD. In addition, OLI-fed pigs showed downregulation of genes involved in neurotransmission, synaptic transmission, and nervous tissue development. Similarly, COC-fed pigs showed upregulation of neurogenesis and myelin repair genes, which caused the accumulation of medium-chain acylcarnitines in brain tissue. In conclusion, our results indicate that secondary BA levels in the FCs of NAFLD pigs are affected by dietary FA composition and are associated with metabolic and transcriptomic markers of brain injury. Dietary interventions that aim to replace saturated FAs by medium-chain or monounsaturated FAs in high-fat hypercaloric diets may have a negative effect on brain health in NAFLD patients.

## 1. Introduction

Non-alcoholic fatty liver disease (NAFLD) is the most common chronic liver disease globally, affecting approximately 25% of the general population [1]. NAFLD is defined as a spectrum of diseases related to hepatic fat deposition, ranging from steatosis to non-alcoholic steatohepatitis (NASH), which can progress to fibrosis, cirrhosis, and hepatocellular carcinoma [2]. In addition to liver injury, NAFLD has been associated with neurological pathologies, including decreased cognitive function [1,3,4,5], and smaller brain volume [4,6]. Further evidence of a link between liver and brain disorders is shown by the development of neuropathological hallmarks of Alzheimer’s disease (AD) in rodent and pig models of NASH [7,8,9] and the presence of altered hepatic markers in patients with AD [10,11]. A liver–brain axis of neurodegeneration has been established through common risk factors, such as obesity, type 2 diabetes, and high-fat diets [7,12,13,14]. In this regard, recent clinical and translational studies have shown an effect of dietary interventions on brain function, such as changes in cognitive processes, memory and learning, motor function, neuroinflammation, synaptic transmission, and neurotransmitter pathways [15,16,17,18]. In addition, emerging evidence suggests that bile acids (BAs) and BA signaling may also play a role in liver-induced brain injury, due to their potential neurotoxic effects [19,20,21] and their ability to regulate neuroinflammation and brain cholesterol metabolism [22,23,24]. Increased levels of circulating BAs have been correlated with the progression of NAFLD in adults, children, and pigs [25,26,27], and have been found to promote hepatic stellate cell proliferation, inflammation, and apoptosis in vitro [28,29]. Similarly, elevated BAs in the brain or serum have been associated with neurodegeneration in mouse models of liver failure [19,22,24,30] and in patients with AD [10,31,32] and hepatic encephalopathy [33]. A connection between BAs and neurodegeneration is further supported by clinical interventions targeting BA signaling, in which BA receptor agonists have been shown to alleviate various aspects of AD and Parkinson’s disease (PD) [34,35,36].

Bile acids are of key importance in the absorption of dietary fats by solubilizing cholesterol and lipids in the small intestine. Reciprocally, BA metabolism can be regulated by fat intake, with fat-free diets decreasing and fat feeding increasing BA synthesis in mice [37,38]. Consumption of a diet high in milk fats also promoted hepatic taurine conjugation of BAs in mice, which in turn caused gut dysbiosis and inflammation [39]. A role of dietary fats in BA kinetics has also been observed in humans, with unsaturated fats increasing total BA output [40,41], and both low- and high-fat diets decreasing primary BA synthesis [42]. Surprisingly, there are no studies assessing the impact of dietary fats on brain BA metabolism in NAFLD patients. We have previously established a pig model of pediatric NAFLD, where Iberian pigs fed a “Western diet” for 10 weeks showed accumulation of primary BAs in the frontal cortex (FC), astrogliosis and neuronal loss, and decreased cognitive function compared with healthy controls [9,25]. In addition, high-fat diets enriched with lard (high in saturated fatty acids (SFAs) and long-chain fatty acids (LCFAs)), olive oil (high in monounsaturated fatty acids (MUFAs) and LCFAs), and coconut oil (high in SFAs and medium-chain fatty acids (MCFAs)) differentially regulated hepatic BA metabolism and NAFLD progression in juvenile pigs [43]. The objective of the present study was to investigate the effects of variations in the degree of saturation and carbon chain length of dietary fats on brain BA metabolism and neuronal number in a pig model of pediatric NAFLD. Our results show that secondary BA levels in the FC of NAFLD pigs are affected by dietary fatty acid (FA) composition and associated with metabolic and transcriptomic markers of brain injury.

## 2. Material and Methods

### 2.1. Animals and Experimental Design

**Figure 1:**
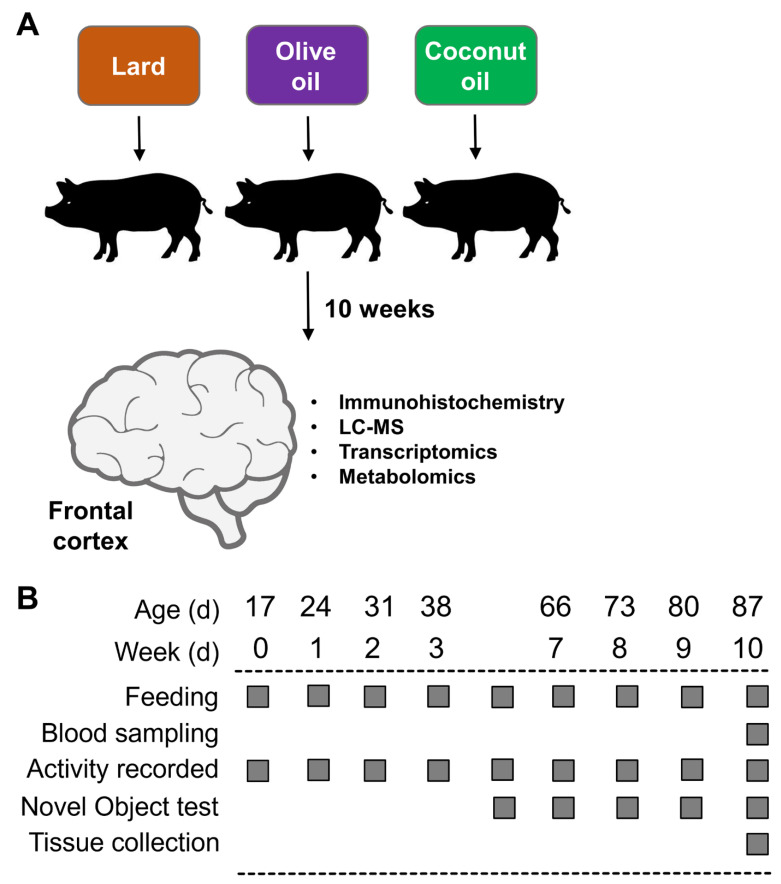
(**A**) Experimental design. (**B**) Timeline of activities during the 10 weeks of the study.

All experiments were carried out with the approval of the Institutional Animal Care and Use Committee of California State University (#1611), following guidelines issued by the National Research Council Guide for the Care and Use of Laboratory Animals. The diets, as well as the characterization of the NAFLD phenotype in the Iberian pigs used in this study, have been described in detail in a recent report [43]. An outline of the study is presented in Figure 1A,B.

Briefly, 19 male (M) and 11 female (F) Iberian pigs from the Iberian Pig Research Colony at California Polytechnic State University were weaned at 17 ± 3 d of age and 4.21 ± 1.21 kg body weight and immediately moved into a temperature-controlled room with a 12 h light–dark cycle. After 3 d of acclimatization, pigs were housed in pairs in 1.5 × 1.5 m pens balanced for sex and BW, and randomly allocated to receive 1 of 3 high-fructose, high-fat liquid diets for 10 consecutive weeks: (1) lard (LAR; *n* = 5 pens, 6M/4F): 21.6 g fructose, 17.2 g fat (8.8% lard, 0.5% soybean oil; *w*/*v*), and 303.0 kcal metabolizable energy (ME) kg·BW^−1^·d^−1^; (2) olive oil (OLI; *n* = 5 pens, 7M/3F): 21.6 g fructose, 16.7 g fat (3.7% lard, 4.8% olive oil, 0.5% soybean oil), and 302.6 kcal·kg·BW^−1^·d^−1^; and (3) coconut oil (COC; *n* = pens, 6M/4F): 21.6 g fructose, 17.0 g fat (3.7% lard, 5% coconut oil, 0.5% soybean oil), and 302.6 kcal·kg·BW^−1^·d^−1^. Complete information about ingredient composition and daily nutrient intake can be found in Manjarin et al., 2022 [43]. The study was conducted in 2 consecutive replicates: replicate 1 consisted of 18 pigs allocated to 9 pens (2 pigs per pen, 3 pens per diet), whereas replicate 2 consisted of 12 pigs allocated to 6 pens (2 pigs per pen, 2 pens per diet).

Animals were fed 45 mL per kg·BW^−1^ at 6 h intervals to match the physiological volume of milk consumed by pigs during lactation. Diets were formulated to meet all the nutrient requirements of growing Iberian pigs according to the NRC [44] and FEDNA [45], and exceeded the NRC recommended daily energy intake by approximately 60%. Protein, carbohydrate, cholesterol, vitamin, and mineral intake were the same across diets. Fat content in LAR was provided by hydrogenated lard, whereas in the OLI diet 58% of lard was replaced isocalorically by extra virgin olive oil to increase MUFA content. Similarly, 58% of lard was substituted isocalorically by hydrogenated coconut oil in the COC diet to increase MCFAs.

The first day on which the experimental diets were fed was considered as d 0 of the study. Animals were euthanized on d 70 using an intramuscular injection of tiletamine and zolazepam (4 mg·kg^−1^; Zoetis, Parsippany, NJ, USA), followed by an intracardiac injection of pentobarbital sodium (0.4 mL·kg^−1^; Schering-Plough, Union, NJ, USA). Brains were removed immediately after euthanasia and weighed. Tissue from the frontal cortex (FC) was washed for 5 s in ice-cold saline solution and frozen in liquid nitrogen or placed in plastic cassettes (Tissue-Tek Cryomold Standard; Sakura, Torrance, CA, USA), covered with optimum cutting temperature compound (cat. no. 4583, Tissue-Tek O.C.T; Sakura, Torrance, CA, USA), and slowly frozen in liquid-nitrogen-cooled 2-methylbutane (cat. no. M0167, TCI, Portland, OR, USA). Tissues were kept at −80 °C until processing. We analyzed the FC tissues based on our previous work, in which juvenile Iberian pigs fed a high-fat, high-fructose diet for 10 weeks developed neuronal loss and astrogliosis in the FC when compared with healthy control animals [9]. In addition, neurons in the FC are among the first to deteriorate in AD patients [46].

### 2.2. Pen Activity and Novel Object Recognition Test

Physical activity in the pen was observed and quantified in the same manner as previously described [9]. In brief, videos were recorded every 2 days between d 16 and 70 of the study from 8:30 AM to 12:30 PM using cameras mounted from the ceiling. Two independent experimenters blinded to the treatment retrospectively scored animals’ activity using Behavioral Observation Research Interactive Software (BORIS; version 7.9) [47] based on the ethogram shown in Table 1. Duration of behavior performance for and the number of pigs performing each behavior were annotated individually for each pen.

Recognition memory was assessed by the novel object recognition test conducted between d 35 and 70 of the study once a week, as previously described [48], because 5-week-old domestic pigs have been shown to remember objects for up to 6 days [49]. In brief, the test was performed 1 h after morning feeding by affixing two identical sample objects to the pen gates with zip-ties to prevent them from being removed and giving the pigs 10 min to explore the objects (sample phase). After 1 h, one sample object and a new object (of the same color but a different shape to that of the sample object) were affixed to the pen gates. Animals were given an additional 10 min to explore the objects (test phase). Two independent experimenters blinded to the treatment retrospectively scored the recorded videos using BORIS, based on the ethogram presented in Table 1. Data are presented as recognition indexes (RIs; time spent investigating novel object/time investigating both objects).

### 2.3. Fatty Acid Composition

Fatty acid composition was quantified by gas chromatography of the FA methyl esters using an Agilent 7890B (Agilent Technologies, Palo Alto, CA, USA) equipped with a flame ionization detector, a 7683B automatic liquid sampler (Agilent Technologies), a split/splitless injection port, and a J&W DB-23 column (Agilent Technologies), with helium used as the carrier gas, as described in our previous work [25]. Values for individual FAs were expressed as peak areas under the curve. Based on FA composition, the following indexes were calculated: saturated FAs (SFAs) = Σ [(%) 8:0 + 10:0 + 11:0 + 12:0 + 13:0 + 14:0 + 15:0 + 16:0 + 17:0 + 18:0 + 20:0 + 21:0 +22:0 +23:0 + 24:0]; unsaturated FAs (UFAs) = Σ [(%) *n*-3 + *n*-5 + *n*-6 + *n*-7 + *n*-9]; monounsaturated FAs (MUFAs) = Σ [(%) *n*-5 + *n*-7 + *n*-9]; polyunsaturated FAs (PUFAs) = Σ [(%) *n*-3 + *n*-6] [50].

### 2.4. Immunofluorescence Analysis

Frontal cortex samples embedded in optimum cutting temperature compound (Sakura) were processed for immunofluorescence staining against a marker for mature neurons, NeuN, as previously described [9]. Briefly, samples were cut, mounted on slides, fixed in cold acetone, washed 3× with phosphate-buffered saline (PBS), and blocked in 2% bovine serum albumin (cat. no. 0332-500G, VWR Life Science, Radnor, PA, USA) and 10% animal-free blocker (cat. no. SP-5030-250, Vector Laboratories, Burlingame, CA, USA) in PBS. Tissue sections were then incubated with a primary antibody against neuronal nuclei (cat. no. MAB377, NeuN, MilliporeSigma, Burlington, MA, USA) in blocking solution. Subsequently, tissues were washed 3× with PBS and incubated with DyLight 594 Horse Anti-Mouse IgG (H+L) (cat. No. DI-2594, Vector Laboratories, Burlingame, CA, USA) in 0.5% bovine serum albumin and 10% animal-free blocker in PBS. Following washing, coverslips were mounted with fluorescence protective medium (cat. no. H-1900, VECTASHIELD Antifade Mounting Medium; Vector Laboratories) and left to dry. Images were taken with a FluoView 500 Confocal Laser Scanning Microscope (Olympus; Center Valley, PA, USA) using a 40× objective by an operator blinded to the treatments. Images were then converted into a z-projection using ImageJ software [51], and the average staining intensity was quantified and reported as a percentage of total area.

### 2.5. Analysis of Metabolites

Primary metabolomic, biogenic amine, lipidomic, and BA assays on frontal cortex samples were performed by protein precipitation extraction with ultra-performance liquid chromatography–tandem quadrupole mass spectrometry, as previously described [25]. Fifty-sixty milligrams of the FC tissue was spiked with 20 µL of isotopically labeled surrogates (Avanti Polar Lipids, Alabaster, AL, USA; CDN Isotopes, Pointe-Claire, Quebec, QC, Canada), followed by 750 µL chilled methanol. Samples were then vortexed for 1 min and centrifuged at 12,000 rpm for 10 min at 4 °C. The supernatant was transferred to 1.5 mL high-performance liquid chromatography amber glass vials, dried by centrifugal vacuum evaporation, and reconstituted in 3:1 methanol:acetonitrile containing 100 nM of 1-cyclohexyl-ureido, 3-dodecanoic acid (MilliporeSigma). The reconstituted solution was vortexed for 1 min and filtered through a polyvinylidene fluoride membrane (Durapore PVDF, 0.1 µm; MilliporeSigma) by centrifugation at 9500 rpm for 3 min at room temperature. Analyses were conducted on a Waters UPLC Acquity I-Class (Waters, Milford, MA, USA) coupled with a 4000 QTRAP LC-MS/MS System (SCIEX, Concord, ON, Canada), using multiple reaction monitoring [52] quantified with MultiQuant Software version 3.0 (SCIEX). General metabolites were separated using a 150 × 2.0 mm Luna NH2 column (Phenomenex, Torrance, CA, USA) and detected by negative ion mode electrospray ionization [52,53]. For the aminomic assay, metabolites were separated using a 150 × 2.1 mm Atlantis HILIC column (Waters) and detected by positive ion mode electrospray ionization [52]. For the lipidomic assay, metabolites were separated using a 150 × 3.0 mm Prosphere HP C4 column (Grace Discovery Sciences, Columbia, MD, USA) and detected by positive ion mode electrospray ionization [52,54]. For BA analysis, analytical targets were separated using a 2.1 × 100 mm, 1.7 µm BEH C18 column (Waters) operated in negative mode electrospray ionization.

Metabolite intensities were normalized to those of internal standards (Cayman Chemical Company, Ann Arbor, MI, and Avanti Polar Lipids) and to sample weights to account for small variations in the starting tissues, and were expressed as peak areas under the curve. Metabolites were quantified using internal standards (Cayman Chemical Company, Ann Arbor, MI, USA; and Avanti Polar Lipids, Alabaster, AL, USA) with 6- to 8-point calibration curves. In addition, BAs were standardized to relative composition (so that sample totals were 100%).

### 2.6. Transcriptomic Analysis

Frontal cortex tissue from 1 pig per pen (5 LAR, 5 OLI, and 5 COC) were sent to GENEWIZ, LLC. (South Plainfield, NJ, USA) for RNA isolation, library preparation, and sequencing. Total RNA was extracted using the RNeasy Plus Mini Kit (cat. no. 74134, Qiagen, Hilden, Germany), quantified using a Qubit 2.0 Fluorometer (Life Technologies, Carlsbad, CA, USA), and assessed for integrity (RIN) using the TapeStation 4200 automated electrophoresis tool (Agilent Technologies, Palo Alto, CA, USA). The preparation of RNA libraries was performed using the NEBNext Ultra RNA Library Prep Kit for Illumina, following the manufacturer’s instructions (cat. no. E7770, NEB, Ipswich, MA, USA). Briefly, mRNA samples were poly(A)-enriched with oligo(dT) beads and heat fragmented, and first- and second-strand cDNAs were synthesized. cDNA fragments were subjected to end repair, 3′-end adenylation, ligation of universal adapters, and the addition of index barcodes. The sequencing libraries were enriched with limited-cycle PCR and validated and quantified with an Agilent TapeStation and a Qubit 2.0 Fluorometer, with additional quantification by quantitative PCR (KAPA Biosystems, Wilmington, MA, USA). RNA libraries were sequenced using a 2 × 150 bp Paired-End configuration of the Illumina HiSeq 4000 system on 2 flow cell lanes. De-multiplexing and conversion of raw sequence data into fastq files were performed using bcl2fastq 2.17 software (Illumina), with one mismatch allowed during index sequence identification.

The swine (*Sus scrofa*) reference genome, SSC11.1, and annotation file (GTF) were downloaded from the Ensembl FTP site (ftp://ftp.ensembl.org/pub/release-100/fasta/sus_scrofa/dna/, accessed on 17 March 2022). The sequence quality of raw paired-end reads was evaluated using FastQC software version 0.11.9 with default parameters (https://www.bioinformatics.babraham. Ac.uk/projects/fastqc/, accessed on 17 March 2022). All the samples passed the quality control by FastQC. Then, reads were aligned to the swine genome Sscrofa11.1 using STAR aligner software (-2.7.5a), with default parameters which are optimized for mammalian genomes [55]. A read-count table showing how many reads mapped to annotated genes was generated by the feature Counts package in Subread software version 2.0.1 [56] using a reference genome sequence, a gene annotation file, and sorted bam files. Reads that were uniquely aligned to each gene annotated in the GTF were counted and used for further analyses, whereas the reads with multi-mapping, no features, or ambiguity were excluded.

### 2.7. Statistical Methods

The pen was considered as the experimental unit for all analyses, except for the transcriptomic analyses, in which each pig was analyzed individually. Univariate data were analyzed by one-way ANOVA using a mixed model in SAS 9.2 (PROC MIXED; SAS Institute Inc., Cary, NC, USA) that included diet as the fixed effect, and both replicate and pen nested in diet as random effects. The normality of the residuals and the presence of outliers were assessed with PROC UNIVARIATE (SAS). Non-normally distributed parameters were power-transformed by a parameter, φ, whose optimal value was estimated using the maximum likelihood method [57]. Data are presented as means ± SDs. Multiple comparisons were corrected with Tukey’s post hoc test, and significant effects were considered at *p* ≤ 0.05. Identification of metabolites differentially expressed between diets was performed using the *%polynova_2way* SAS macro, as previously described [58]. Metabolomics data were further assessed by principal component analysis (PCA) to visualize group discrimination in a two-dimensional scores plot. PCA analyses were conducted in R Statistical Language version 4.1.0. Missing data were imputed using the K-Nearest-Neighbor imputation algorithm [59]. Metabolite peak areas were log-transformed and scaled to unit variances prior to PCA.

The statistical analyses of differentially expressed genes (DEGs) were performed using the edgeR-Bioconductor packageBio package [60] in R software. First, the non-expressed and very lowly expressed genes were filtered out, keeping genes that were expressed at a reasonable level (counts per million (CPM) > 0.5). To account for the variation due to library sequencing depths between samples, the read counts were normalized using the trimmed means of M values (TMM) method implemented in edgeR. The read counts were then analyzed with a generalized linear model, with an assumption of a negative binomial distribution of gene counts to identify differentially expressed genes between treatments. The statistical model used for analyses was as follows: log (CPM)_ijk_ = μ + treatment_i_ + e_ijk_), where the log (CPM)_ijk_ is the log-transformed read CPM of mapped reads for the gene k in sample j from the ith treatment group, μ is the effect of the intercept or the expected (average) gene expression, and e_ijk_ is the random residual error effect. A likelihood ratio test for each gene expression level between the treatment groups was used to identify the DEGs. To adjust *p*-values for multiple testing, the false discovery rate (FDR) method was used, where the significant differentially expressed genes were determined using a 5% FDR threshold. The functional enrichment analyses were performed on DEGs (at *p* ≤ 0.05) to identify GO terms using the Database for Annotation, Visualization and Integrated Discovery (DAVID) software version 6.8 [61]. The expression of DEGs associated with relevant GO terms was represented in heatmaps constructed with ClustVis software (BETA) [62].

Associations between metabolites and DEGs were identified using a multivariate application of sparse partial least squares (sPLS) [63]. The adequate number of features to retain were calculated by optimizing the average correlation coefficients between latent variables using 10-fold cross-validation with 10 repeats. The final sPLS model was fitted using least absolute shrinkage and selection operator (LASSO) penalty to fit the required features. A bipartite pair-wise similarity matrix was then calculated from the sPLS using a threshold criterion of 0.7. The similarity matrix represented robust approximations of Pearson’s correlations [64] and was visualized using network analysis (Cytoscape software version 3.9.0) [65].

## 3. Results

### 3.1. COC and OLI Pigs Had Decreased Physical Activity without Changes in Cognitive Function or Neuronal Loss

The objective of this study was to investigate the effect of fat sources with different FA profiles on neurodegeneration in a pig model of NAFLD using immunofluorescence and behavioral tests combined with multiomic analyses of FC tissue. The characterization of the NAFLD phenotype in the Iberian pigs used in this study, including biochemistry, histology, metabolomics, and transcriptomics in the liver, blood, and gut, has been described in detail in Manjarin et al. 2022 [43]. Briefly, steatotic grade (*p* ≤ 0.05), necrosis (*p* ≤ 0.01), hepatocellular proliferation (*p* ≤ 0.05), and composite lesion score (*p* ≤ 0.01) in liver tissues were higher in COC and OLI compared with LAR (Table 2). Similarly, serum biochemistry showed an increase in alanine transaminase (*p* ≤ 0.05), aspartate transaminase (*p* ≤ 0.05), and lactate dehydrogenase (*p* ≤ 0.05) in COC and OLI, while gamma-glutamyl transferase was elevated in COC-fed pigs compared with LAR (*p* ≤ 0.05; Table 2).

**Figure 2:**
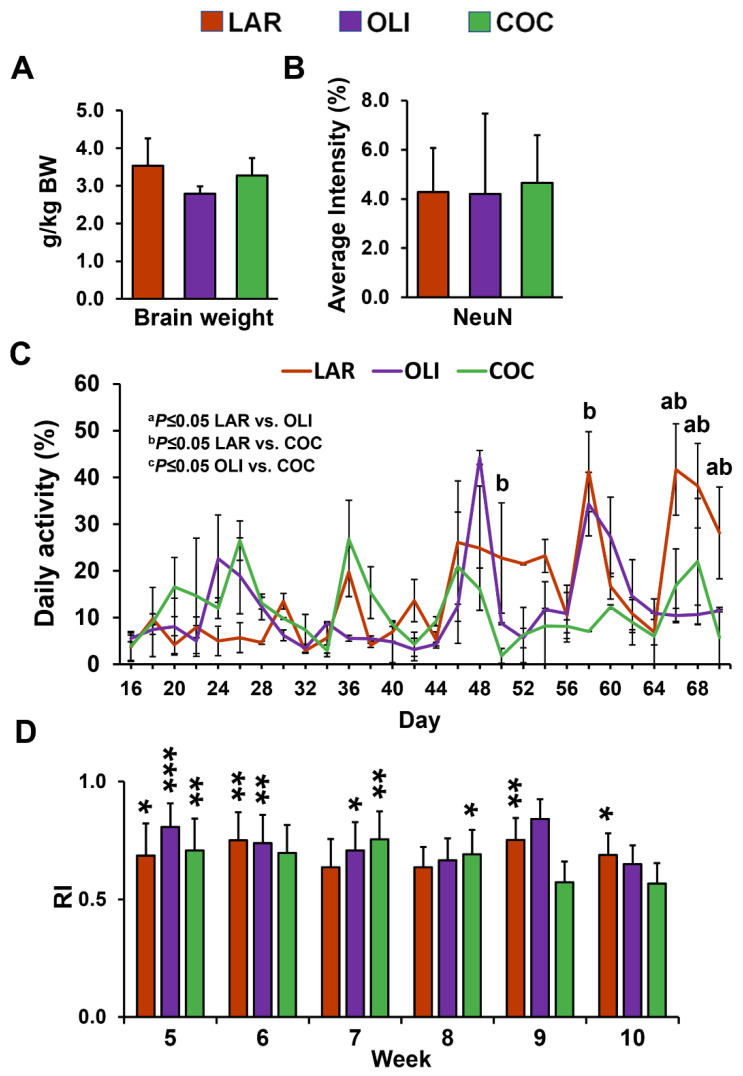
(**A**) Relative brain weight expressed as grams of brain tissue per kg of body weight. (**B**) Quantification of staining intensity of neuronal nuclei (NeuN) in the frontal cortex expressed as a percentage of the total area. (**C**) Pen activity, measured daily between d 1 and 70 of the study from 8:30 AM to 12:30 PM. Values are means ± SDs. (**D**) Histograms representing results for the recognition index (RI). The indexes were calculated based on the formula (time spent investigating novel object/time investigating both objects). Significant *p*-values for daily activity were adjusted for multiple testing with Tukey post hoc tests and expressed as ^a^ *p* ≤ 0.05, ^b^ *p* ≤ 0.05 LAR vs. COC, and ^c^ *p* ≤ 0.05 OLI vs. COC. *p*-values for one-tailed *t*-tests significantly different from 0.5 are expressed as * *p* ≤ 0.05, ** *p* ≤ 0.01, *** *p* ≤0.001. COC, coconut oil diet; LAR, lard diet; OLI, olive oil diet.

No differences were observed in relative brain weight and neuronal marker NeuN immunofluorescence among the diet groups (Figure 2A,B). Next, we assessed changes in physical activity and cognitive functioning. The mixed-model ANOVA indicated a significant increase in daily activity levels in LAR compared with COC on d 50 and 58 (*p* ≤ 0.05), and in COC and OLI on d 66, 68, and 70 (*p* ≤ 0.05; Figure 2C). There were no differences in the novel object recognition test between weeks 5 and 10 across treatment groups (Figure 2D). In addition, RI was significantly greater than 0.5 for all diets at week 5 (*p* ≤ 0.05), for LAR and OLI at week 6 (*p* ≤ 0.01), OLI and COC at week 7 (*p* ≤ 0.05), COC at week 8 (*p* ≤ 0.05), and LAR at weeks 9 and 10 (*p* ≤ 0.05; Figure 2D).

### 3.2. COC and OLI Diets Decreased Secondary BA Species Compared with LAR

**Figure 3:**
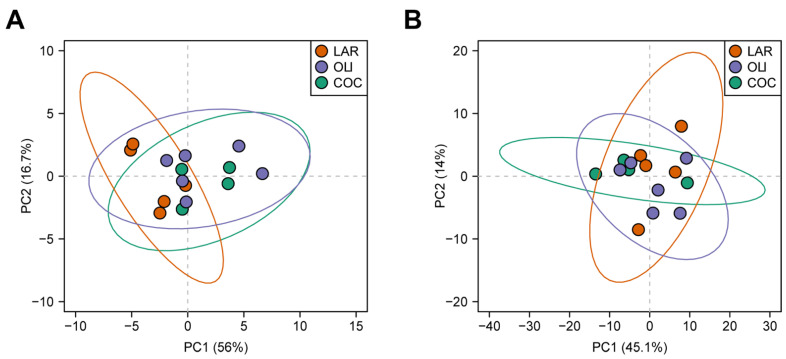
Principal component analysis of bile acids (**A**) and metabolites (**B**) in the frontal cortexes of juvenile Iberian pigs. Data were scaled to unit variance prior to PCA assessment. Two-dimensional visualizations of PCA scores are projected from their group centroids along components 1 and 2. *p*-, R-squared, and F-statistic values are derived from ANOVA assessed on the first principal component. Each point represents an individual pig; the color of the point denotes the diet.

Twenty BA species were detected in the FCs of juvenile Iberian pigs. Principal component analysis separated LAR from OLI and COC, but could not separate OLI and COC samples (*p* ≤ 0.05; Figure 3A).

**Figure 4:**
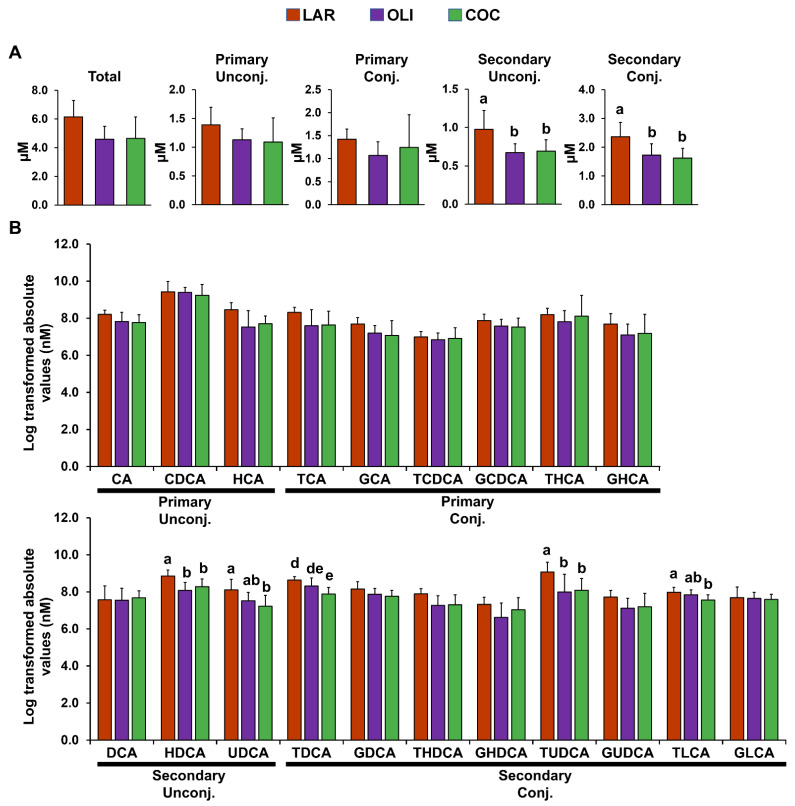
(**A**) Total, primary, and secondary levels of BAs in the frontal cortex of juvenile Iberian pigs fed LAR (*n* = 5 pens), OLI (*n* = 5 pens), and COC (*n* = 5 pens) diets on day 70 of the study. (**B**) Abundance of individual BAs in the frontal cortex. *p*-values for each metabolite were calculated by one-way ANOVA and a mixed model that included diet as fixed effect, and both replicate and pen nested in diet as random effects, and further adjusted for multiple testing with the Benjamini–Hochberg procedure. Values are means ± SDs. *p*-values were adjusted for multiple testing with Tukey’s post hoc test. ^ab^ *p* ≤ 0.05, ^de^ *p* ≤ 0.01. CA, cholic acid; CDCA, chenodeoxycholic acid; COC, coconut oil diet; DCA, deoxycholic acid; G, glycine conjugated; HCA; hyocholic acid; HDCA, hyodeoxycholic acid; LAR, lard diet; LCA, lithocholic acid; OLI, olive oil diet; T, taurine conjugated; UDCA, ursodeoxycholic acid.

Compared with LAR, secondary conjugated and unconjugated BAs in the FC decreased in COC and OLI (*p* ≤ 0.05; Figure 4A). Analysis of individual secondary unconjugated BA species showed a decrease in hyodeoxycholic (HDCA) and ursodeoxycholic (UDCA) acids in OLI and COC compared with LAR (*p* ≤ 0.05; Figure 4B). Among secondary conjugated BAs, taurine-conjugated deoxycholic (TDCA) and lithocholic (TLCA) acids decreased in COC (*p* ≤ 0.01 and 0.05, respectively; Figure 4B), and TUDCA decreased both in COC and OLI compared with LAR (*p* ≤ 0.05; Figure 4B).

### 3.3. COC and OLI Decreased One-Carbon Metabolites, Amino Acids, Complex Lipids, and Carboxylic Acids in the Frontal Cortex

**Figure 5:**
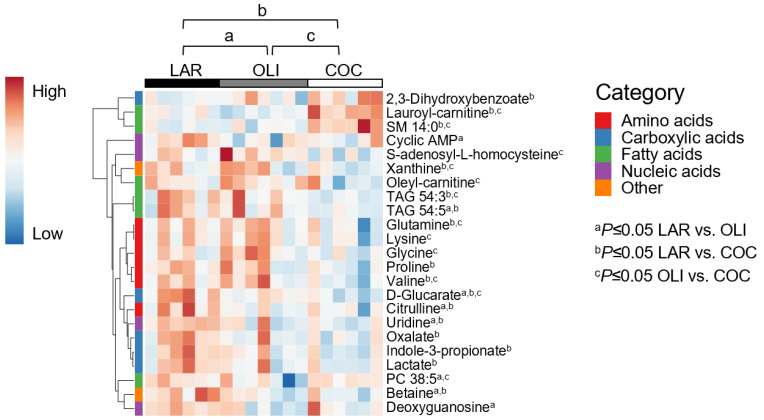
Heat map of absolute abundance of metabolites significantly altered by diet in the frontal cortexes of juvenile pigs, measured by ultra-performance liquid chromatography–tandem quadrupole mass spectrometry. Columns represent individual pigs and rows represent log_2_-transformed metabolites. Blue and red colors represent the row minimum and maximum values, respectively. *p*-values for each metabolite were calculated by one-way ANOVA and a mixed model that included diet as fixed effect, and both replicate and pen nested in diet as random effects, and were adjusted for multiple testing with the Benjamini-Hochberg procedure. Values are means ± SDs. *p*-values were adjusted for multiple testing with Tukey´s post hoc test. ^a^ *p* ≤ 0.05, ^b^ *p* ≤ 0.05 LAR vs. COC, and ^c^ *p* ≤ 0.05 OLI vs. COC. COC, coconut oil diet; LAR, lard diet; OLI, olive oil diet.

A total of 127 metabolites were detected in the FC, of which 23 changed between diets. PCA plots did not separate LAR, OLI, and COC samples (Figure 3B). The amino acids (proline, valine, glutamine, lysine, and glycine), carboxylic acids (oxalate, D-glucarate, indole-3-propionate, and lactate), complex lipids (oleyl-carnitine and TAG 54:3), S-adenosyl-L-homocysteine, and xanthine decreased (*p* ≤ 0.05) in COC compared with OLI and/or LAR (Figure 5). In addition, citrulline, TAG 54:5, betaine, and uridine decreased (*p* ≤ 0.05) in COC and OLI compared with LAR, and PC38:5, cyclic AMP, and deoxyguanosine decreased (*p* ≤ 0.05) in OLI compared with COC and LAR (Figure 5). Conversely, 2-3-dihydroxybenzoate, lauroyl-carnitine, and SM 14:0 increased (*p* ≤ 0.05) in COC compared with OLI and/or LAR (Figure 5).

The major FAs in the FC homogenates were palmitic (16:0), stearic (18:0), oleic (18:1), and arachidonic (20:4) acids (Table 3). No significant differences were observed in FA composition in the FC among LAR, OLI, and COC pigs (Table 3).

### 3.4. COC and OLI Diets Regulated Genes Associated with Myelin Formation, Neuronal Development, and Signaling

**Figure 6:**
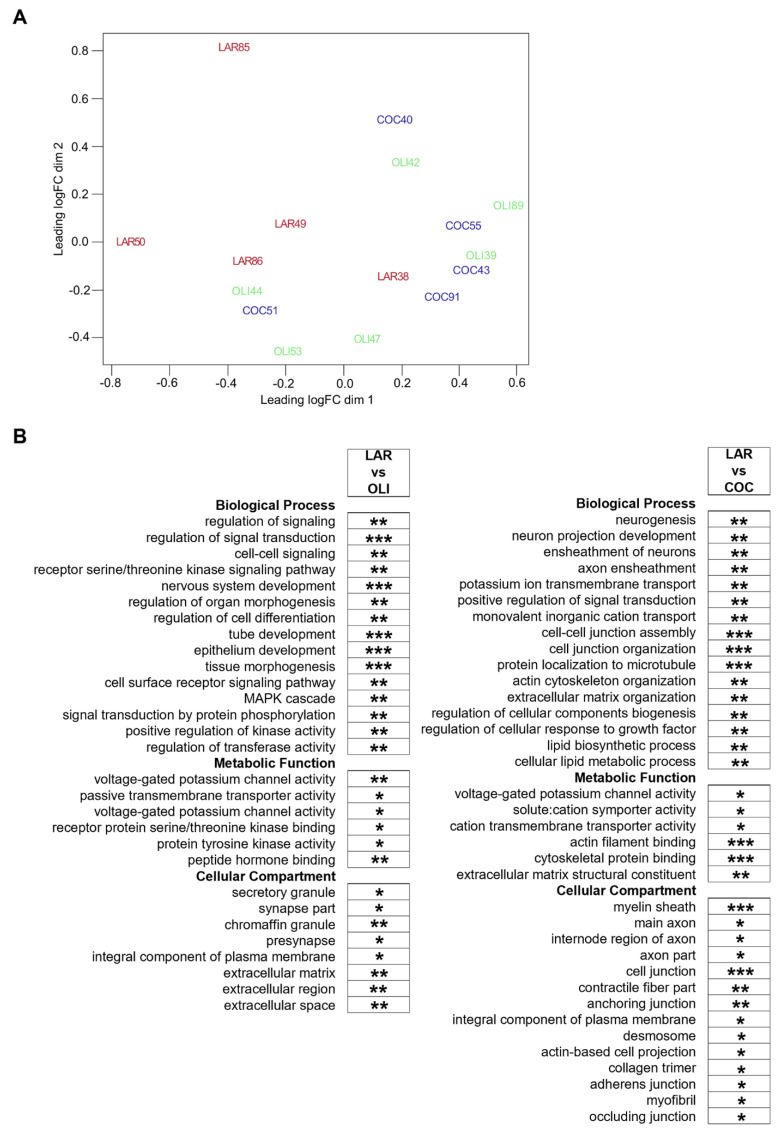
(**A**) Multidimensional scaling diagram of FC samples indicating the groupings obtained from the multivariate analysis. (**B**) Functional enrichment analyses performed on differentially expressed genes at 5% FDR to identify GO terms pertaining to Biological Process, Metabolic Function, and Cellular Compartment in the frontal cortexes of juvenile Iberian pigs using the Database for Annotation, Visualization and Integrated Discovery software version 6.8. * *p* ≤ 0.05, ** *p* ≤ 0.01, *** *p* ≤ 0.001. COC, coconut oil diet; FDR, false discovery rate; LAR, lard diet; OLI, olive oil diet.

Diet-induced changes in the FCs of COC- and OLI-fed pigs were further evaluated through transcriptome-wide RNA profiling. Multidimensional scaling showed the association of FC samples in two differentiated clusters in the chart, with LAR pigs separated from OLI and COC (Figure 6A). Functional enrichment analyses of LAR vs. COC identified terms associated with neurogenesis, myelin formation, voltage-gated channels, organization of extracellular matrix (ECM) and cytoskeleton, and cell adhesion (*p* ≤ 0.05; Figure 6B). In OLI brains, functional enrichment analyses showed deregulation of GO terms related to cell signaling, nervous and epithelial tissue development, voltage-gated channels, synapses, and ECM when compared with LAR (*p* ≤ 0.05; Figure 6B).

**Figure 7:**
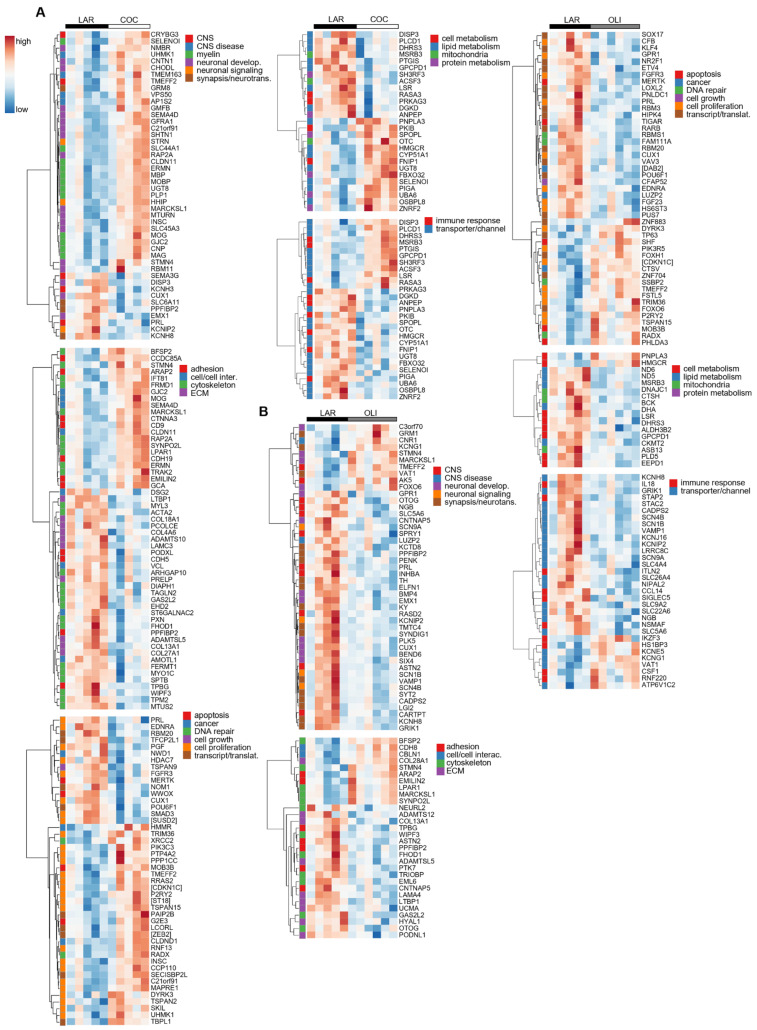
Heat maps of differentially expressed genes (5% FDR) between LAR and COC (**A**) and LAR and OLI (**B**) in the frontal cortex of juvenile Iberian pigs on d 70 of the study. RNA libraries were sequenced using a 2 × 150 bp Paired-End configuration of the Illumina HiSeq 4000 system on two flow cell lanes. The read counts were normalized using the trimmed means of M values (TMM) method implemented in edgeR and then analyzed with a generalized linear model, with an assumption of a negative binomial distribution. Columns represent individual pigs and rows represent log-transformed read counts per million for each gene. Blue and red colors represent the row minimum and maximum values, respectively. COC, coconut oil diet; FDR, false discovery rate; LAR, lard diet; OLI, olive oil diet.

The transcriptional profile highlighted the upregulation (*p* ≤ 0.05; Figure 7A) of many genes in COC brains associated with the formation, maintenance, and structure of the myelin sheath, such as Myelin Oligodendrocyte Glycoprotein (*MOG*)*,* Myelin-Associated Oligodendrocyte Basic Protein (*MOBP*)*,* Myelin Basic Protein (*MBP*), and Myelin-Associated Glycoprotein (*MAG*). Gene sets related to the differentiation, migration, and regeneration of brain cells, such as Glia Maturation Factor Beta (*GMFB*), Shootin 1 (*SHTN1*), Glial-Cell-Derived Neurotrophic Factor Family Receptor Alpha 1 (*GFRA1*), Semaphorin 4D (*SEMA4D*), Neural Progenitor Differentiation Regulator (*MTURN*), Neuromedin B Receptor (*NMBR*), and Contactin 1 (*CNTN1*) were also upregulated in COC compared with LAR (*p* ≤ 0.05; Figure 7A). Conversely, transcripts associated with the ECM, including collagen subunits (*COL13A1*, *COL28A1*, *COL4A6*, *COL18A1*, and *COL27A1*) and Metalloprotease-like Enzymes that modulate microfibril assembly (*ADAMTS10* and *ADAMTSL5*), decreased in COC compared with LAR (*p* ≤ 0.05; Figure 7A).

In OLI-fed pigs, the transcriptional profile showed the downregulation (*p* ≤ 0.05; Figure 7B) of genes coding for voltage-gated sodium and potassium channels (*SCN9A*, *SCN1B*, *SCN4B*, and *KCNIP2*), as well as genes associated with the development and activity of synapses and neurotransmitters, such as Polypeptide-Interacting Protein-Binding Protein 2 (*PPFIBP2*), Proenkephalin (*PENK2*), Extracellular Leucine Rich Repeat And Fibronectin Type III Domain Containing 1 (*ELFN1*), Synapse Differentiation Inducing 1 (*SYNDIG1*), and Glutamate Ionotropic Receptor Kainate Type Subunit 1 (*GRIK1*). Gene sets related to cancer, cell division, cell growth and transcription/translation, coagulation, immune response, and lipid and protein metabolism were also differentially regulated in the FCs of the COC and OLI groups compared with LAR (*p* ≤ 0.05; Figure 7A,B), but were not consistently up- or downregulated nor significantly associated with GO terms in the FC.

### 3.5. Secondary Bile Acids Were Correlated with Expression of Frontal Cortex Genes Involved in Neurogenesis, Neurotransmission, and Extracellular Matrix Organization

**Figure 8:**
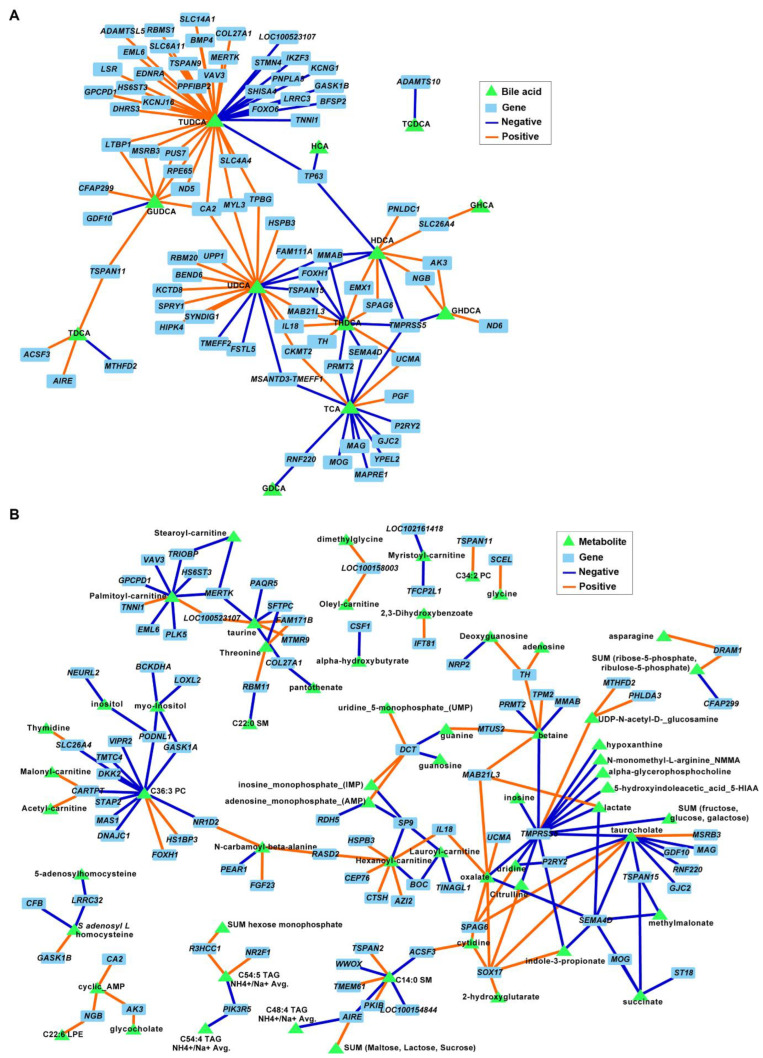
Associations between differentially expressed genes (DEGs) and bile acids (**A**) and metabolites (**B**) in the frontal cortexes of juvenile Iberian pigs fed LAR (*n* = 5 pens), OLI (*n* = 5 pens), and COC (*n* = 5 pens) diets on day 70 of the study. Network displays robust approximations of Pearson’s correlations using sparse partial least square (sPLS) with LASSO penalization. Orange and blue edges represent positive and negative correlations, respectively.

The correlation analysis between BAs and DEGs in FC tissues revealed an association between both TUDCA and UDCA and several genes involved in neuronal development and ECM organization (*p* ≤ 0.001; Figure 8A). For example, negative associations were identified between TUDCA and neurogenesis, neurotransmission, and synapse development genes *STMN4*, *FOXO6*, *BMP4*, *PPFIBP2*, *SLC6A11*, and *KCNG1*, and between TUDCA and ECM genes *COL27A1* and *ADAMTSL5*. Similarly, UDCA was correlated with genes involved in nervous system functions (negative: *SPRY1* and *KCTD8*; positive: *BEND6* and *TMEFF2*). Several metabolites were also associated with DEGs in the FC; however, none of the correlation nodes involved metabolites that were significantly different between diets and/or genes associated with relevant cell functions (Figure 8B).

## 4. Discussion

The main objective of this study was to investigate the effect of fat sources with different FA profiles on brain BA metabolism and neuronal loss in a pig model of NAFLD using a multiomic approach combined with immunohistochemistry and behavioral analyses. In a completely randomized design with two consecutive replicates, 30 juvenile Iberian pigs were fed one of three high-fat, high-fructose diets enriched with lard (high in SFAs and LCFAs), olive oil (high in MUFAs and LCFAs), and coconut oil (high in SFAs and MCFAs). Our results show that the COC and OLI diets lowered secondary conjugated and unconjugated BAs in the FCs of NAFLD pigs, reduced physical activity, and altered expression of metabolic and transcriptomic markers of brain injury. In addition, diet-induced changes in FC metabolites and genes paralleled the severity of NAFLD, as both COC and OLI diets increased steatosis, necrosis, and cellular proliferation in the liver compared with LAR [43]. However, recognition memory and neuronal number did not differ between diets, suggesting that metabolic and transcript abnormalities may precede more severe functional or histopathological changes in the cortex. Interestingly, NeuN average staining intensities in the FCs of the COC, LAR, and OLI groups were similar to the NeuN intensity in the FCs of high-fructose, high-fat-diet-fed juvenile Iberian pigs with neurodegeneration [9], suggesting that the three high-fat diets may have promoted some degree of neuronal loss in the animals when compared with healthy controls.

Many preclinical studies have demonstrated a neuroprotective role for secondary BAs in the brain [66]. For example, TLCA, which was lower in COC-fed pigs compared with the LAR group, decreased microglial production of interleukins 1β and 6 in an LPS model of neuroinflammation [67]. Similarly, administration of HDCA prevented apoptosis and necrosis in astrocytes and neurons exposed to oxygen–glucose deprivation in vitro [68]. Particularly interesting in our study is the decrease in UDCA and TUDCA in COC- and OLI-fed pigs, given that both BAs have been shown to prevent apoptosis, oxidative stress, and inflammation in ex vivo, in vitro, and in rodent models of AD and PD [66]. Furthermore, UDCA is a Farnesoid X receptor antagonist [69], and inhibition of Farnesoid X signaling in the FC has been shown to protect against toxic cholesterol accumulation in mouse models of hepatic encephalopathy [23]. UDCA is produced by gut bacteria through the deconjugation of primary BAs in the distal ileum and colon [1]. Most UDCA is then excreted in the feces, with a small fraction being reabsorbed in the colonic mucosa and circulated into the liver with the portal blood. Once in the liver, UDCA is reconjugated with taurine (t) or glycine (g) before being released again into the small intestine [1]. It is thought that most UDCA and TUDCA in the brain comes from peripheral circulation, as both BAs can be detected in blood and brain and can penetrate across the blood–brain barrier and the cerebrospinal fluid [70,71,72]. However, levels of conjugated and unconjugated UDCA did not decrease in the livers, sera, or colons of COC- or OLI-fed pigs [43], suggesting an alteration of BA transport in the brain rather than systemic BA dysregulation. Similar results were observed in our previous study, in which high-fat-diet-fed pigs with NAFLD and neuronal loss had decreased levels of UDCA and TUDCA in the brain but not in the liver or serum [9,25]. A possible explanation for this effect could be attributed to the decrease in circulating FGF19 levels in OLI and COC animals [43], which has been shown to downregulate the BA transporters OATP and NTCP in the liver and to decrease BA uptake [73]. In this regard, OATP and NTCP have also been detected at the blood–brain barrier in humans and rats and have been shown to participate in UDCA and TUDCA transport [74,75].

We also observed a decrease in one-carbon metabolites [76], amino acids [77], and lactate [78] in the FCs of COC- and OLI-fed pigs compared with LAR. The depletion of brain levels of glutamine and creatinine suggests alterations in neuronal nitric oxide production, which have been associated with the development of AD [79,80,81]. Similarly, decreases in cerebral lactate contents were correlated with reduced amounts of neurons and oligodendrocytes and increased quantities of astrocytes in a mouse model of AD [78]. Of particular importance in the context of brain injury is the decrease in betaine levels in OLI- and COC-fed pigs. Changes in betaine levels in the brain occur as a result of betaine being converted back to choline, which is required for neurotransmitter synthesis and lipid metabolism in neurons [82,83]. In addition, betaine can serve as a methyl donor for the homocysteine-to-methionine reaction in one-carbon metabolism [82,83]. As such, a decrease in betaine levels in COC- and OLI-fed pigs may have altered choline, homocysteine, and gene methylation levels in the FC, with potential neurodegenerative effects [84,85,86]. In this regard, we have previously shown a disproportionate decrease in FC levels of betaine in NAFLD pigs which was positively correlated with astrogliosis and neuronal loss [9]. Given that betaine levels were also lower in OLI and COC livers compared with LAR [43], it is possible that OLI and COC diets caused a systemic dysregulation of choline metabolism. In agreement with this idea, we have previously shown that our pediatric pig model resembles choline-deficient dietary models of NASH, in which pigs develop steatosis, lobular inflammation, and ballooning in the absence of other metabolic features seen in human NAFLD [9,25].

Diets high in total and saturated fats are known to induce brain inflammation, immune cell infiltration, and mitochondrial alterations in mice [87,88,89] and deterioration of cognitive functions in healthy patients [90,91]. Conversely, consumption of the MUFA-enriched Mediterranean diet has been associated with protection against cognitive decline [92,93,94] and reduced neurological dysfunction in rodent models of AD [1,95,96,97]. However, OLI-fed pigs (high in MUFAs) showed downregulation of genes involved in neurotransmission, synaptic transmission, and nervous tissue development, which have been linked to neurological disorders [98,99]. These contradictory results are likely due to the elevated amount of olive oil in the OLI diet (>100 g/d in 20 kg pig), as short-term consumption of low doses of extra virgin olive oil (26 g/d) had a neuroprotective effect in elderly patients [92], whereas high intake of MUFAs (42 g/d) was associated with decreased learning and memory abilities in younger women [100]. Similarly, high MUFA consumption was predictive of mild cognitive impairment in 60- to 64-year-old patients assessed over 4 years [101]. It has been suggested that excessive MUFA intake may have detrimental effects on cognitive function via regulating inflammatory markers and inflammatory signaling pathways in the brain. For example, feeding mice an obesogenic diet with 45% total kcal as oleic acid instead of palmitic acid predisposed neurons and microglia towards an inflammatory phenotype ex vivo [102]. In a separate study, mice fed a high-fat diet enriched with MUFAs showed decreased learning and memory in parallel with the upregulation of interleukin 6 and TLR-MyD88-NF-κB inflammatory signaling pathways in the brain [103]. Interestingly, levels of oleic acid and expression of genes associated with inflammatory cytokines and immune response did not differ between OLI and LAR. Similarly, we did not observe changes in the FC cytokine levels or microglia activation in response to high-fat-diet intake in our previous work [9]; therefore, the etiology of brain transcriptome changes in OLI-fed pigs remains unknown. We cannot discard the possibility that these differences were due to the duration of the study or diet composition. In this regard, previous work has shown an amelioration of NAFLD following inhibition of microglia [104,105].

In addition to the degree of saturation, there is evidence to suggest that carbon chain length is a major determinant of the metabolic effects of dietary FAs in the brain. Substitution of LCFAs with MCFAs, by replacing sunflower oil with medium-chain triacylglyceride (MCT) oils, had a positive effect on cognition and markers of synapse formation in rats fed a weight-maintenance diet [106]. Similarly, isocaloric replacement of LCFAs derived from tallow by MCTs promoted cognition-improving effects in non-obese dogs [107]. Data from clinical trials also suggest that MCTs improved cognitive abilities in patients with AD [108,109]. However, a positive role for FA saturation and chain length in brain function is less clear in high-fat hypercaloric diets, with several studies resulting in negative findings associated with MCT intake. Haghikia et al. [110] reported a decrease in the integrity of the blood–brain barrier in mice fed a high-fat diet enriched with lauric acid [110]. A coconut oil high-fat diet was also found to cause neurotoxicity and impair learning and memory ability in obese mice through upregulation of inflammatory signaling pathways in brain tissue [103]. Moreover, short-term ingestion of high levels of coconut oil margarine instead of lard decreased hypothalamic serotonin in mice [111]. The results from our study demonstrate that partial replacement of lard with an isocaloric amount of coconut oil increased the expression of many remyelination genes in the FC, which are upregulated after injuries to the CNS, such as acute demyelination episodes in neurodegenerative diseases [112,113,114]. In addition, compared to LAR, the COC diet downregulated the expression of genes and enzymes involved in ECM organization, including several collagen subunits, which have also been associated with brain injury in mice [115]. Moreover, we report the accumulation of medium-chain acylcarnitines in brain tissue compared with LAR and OLI. Since coconut oil is rich in lauric acid, COC brains may have been overloaded with an excess of medium-chain FAs, resulting in incomplete FA oxidation and subsequent accumulation of lauryl-acylcarnitine in the FC [116], with potential neurotoxic effects [110]. Limitations of the current study are associated with the experimental design and analysis. Most pens in our study contained a male and a female, and therefore it was not possible to address the influence of sexual dimorphism in diet response. In addition, we did not analyze the hippocampus or hypothalamus, which are also studied in diet-induced neurodegeneration research. Finally, we did not investigate microglia and cytokines in FC tissues, as our previous work did not show changes in these parameters in response to high-fat diets.

## 5. Conclusions

In conclusion, our results showed a decrease in neuroprotective secondary BAs and betaine levels in the FCs of OLI and COC pigs which paralleled the severity of NAFLD, suggesting a link between liver and brain disease. In addition, we have also shown a direct effect of dietary FA composition on the expression of genes linked to brain injury, as the COC diet upregulated markers of myelin repair, whereas the OLI diet downregulated genes involved in synapse development and cell signaling. Nutritional recommendations for NAFLD patients suggest limiting fat intake and a reduction in LCFA consumption in favor of MUFAs and MCTs. While this approach has been shown to be beneficial in balanced diets, our findings do not support replacing large amounts of SFAs by MCFAs and MUFAs in high-fat hypercaloric diets, as this may have a negative effect on brain health.

## Figures and Tables

**Table 1:** Ethograms for novel object recognition test and pig activity in the home pens.

Behavior	Description
Pig activity	
Standing	Both pigs in the pen are standing on four legs
Resting	Both pigs in the pen are lying down on the floor
Standing/resting	One pig is standing and one pig is lying down
Novel object recognition test	
Learning phase	
Sample Object I	Pig exploring sample object on left side
Sample Object II	Pig exploring sample object on right side
Memory phase	
Sample Object	Pig exploring sample object on left side
Novel Object	Pig exploring novel object on right side

**Table 2:** Serum biochemistry and quantitative assessment of hepatic histology in juvenile Iberian pigs fed LAR, OLI, and COC diets for 10 consecutive weeks. COC, coconut oil diet; LAR, lard diet; OLI, olive oil diet.

Item ^1^	LAR	OLI			COC		
Nº pigs (pen)	10 (5)	10 (5)			10 (5)		
Sex (M/F)	6/4	7/3			6/4		
Liver histology ^2^							
Steatosis	2.90 ^a^ ± 0.32	3.40 ^ab^ ± 0.52			3.50 ^b^ ± 0.53		
Ballooning	0 ± 0	0.40 ± 0.52			0.30 ± 0.48		
Mallory–Denk Bodies	0.10 ± 0.32	0.20 ± 0.42			0.30 ± 0.48		
Inflammation	1.20 ± 0.42	1.20 ± 0.42			1.33 ± 0.42		
Necrosis	0 ^d^ ± 0	1.00 ^e^ ± 0.47			0.70 ^e^ ± 0.58		
Ki67^+^ cells ^3^	8.23 ^a^ ± 3.45	9.56 ^ab^ ± 3.23			14.7 ^b^ ± 6.85		
Composite lesion score	4.20 ^d^ ± 0.42	6.20 ^e^ ± 1.23			6.00 ^e^ ± 0.82		
Serum biochemistry							
Alanine aminotransferase, U·L^−1^	34.3 ^a^ ± 3.5	70.6 ^b^ ± 18.0			62.2 ^b^ ± 40.4		
Aspartate aminotransferase, U·L^−1^	65.7 ± 48.5	180.5 ± 75.6			199.6 ± 149.2		
Alkaline phosphatase, U·L^−1^	292.6 ± 80.7	387.7 ± 120.8			391.6 ± 149.2		
γ-glutamyl transferase, U·L^−1^	33.3 ^a^ ± 9.9	37.3 ^ab^ ± 4.8			56.8 ^b^ ± 29.4		
Lactate dehydrogenase, U·L^−1^	1819.8 ^a^ ± 1078.3	3986.5 ^b^ ± 829.1			3114.2 ^b^ ± 1492.7		
Total bilirubin, mg·dL^−1^	0.04 ± 0.04	0.02 ± 0.02			0.05 ± 0.03		

^1^ Data are means ± SDs. ^2^ Steatosis: 0 (absent), 1 (<10%), 2 (10–25%), 3 (26–50%), 4 (>50%); ballooning, Mallory–Denk Bodies, fibrosis, inflammation, and necrosis: 0 (absent), 1 (minimal), 2 (mild), 3 (moderate), 4 (severe); composite lesion score: sum of all histological scores. ^3^ Ki67^+^: percentage of proliferative cells in liver. *p*-values were calculated by one-way ANOVA and adjusted by post hoc Tukey test. Labeled means without a common letter differ: ^abc^ *p* ≤ 0.05, ^def^ *p* ≤ 0.01.

**Table 3:** Fatty acid (FA) compositions in the frontal cortexes of pigs fed LAR, OLI, and COC diets. Samples were analyzed by gas chromatography and values expressed as absolute FA composition. Values are means ± SDs. COC, coconut oil diet; FA, fatty acid; LAR, lard diet; LCFA, long-chain FA; MCFA, medium chain FA; OLI, olive oil diet; SFA, saturated FA; UFA, unsaturated FA; MUFA, monounsaturated FA; PUFA, polyunsaturated FA; VLCFA, very-long-chain FA.

		LAR	OLI	COC
FAs				
Caprylic	C8:0	515.8 ± 77.2	514.1 ± 69.3	497.5 ± 56.3
Myristic	C14:0	620.1 ± 63.0	621.6 ± 76.8	664.0 ± 35.3
Pentanedecanoic	C15:1 (*n-5*)	1122.3 ± 206.4	1134.8 ± 125.1	1267.1 ± 259.2
Palmitic	C16:0	7705.1 ± 721.1	7741.0 ± 520.6	7935.6 ± 774.3
Palmitoleic	C16:1 (*n-7*)	587.2 ± 49.0	585.8 ± 49.1	591.7 ± 38.8
Heptadecanoic	C17:0	371.8 ± 32.9	392.8 ± 84.2	357.8 ± 27.7
Heptadecenoic	C17:1 (*n-7*)	302.1 ± 22.8	309.5 ± 45.9	295.5 ± 29.5
Stearic	C18:0	7470.6 ± 930.5	7277.3 ± 678.8	7682.3 ± 1207.3
Oleic	C18:1 (*n-9*)	6305.8 ± 493.6	6572.8 ± 769.5	6559.4 ± 1388.3
Linoleic	C18:2 (*n-6*)	473.1 ± 22.8	472.5 ± 78.2	449.2 ± 41.9
Arachidic	C20:0	617.2 ± 87.8	576.8 ± 67.6	559.1 ± 35.4
Eicosenoic	C20:1 (*n-9*)	478.3 ± 37.6	469.9 ± 43.3	449.4 ± 49.2
Eicosadienoic	C20:2 (*n-6*)	524.7 ± 74.2	573.9 ± 58.4	578.6 ± 78.3
Heneicosanoic	C21:0	323.5 ± 27.9	305.5 ± 20.1	323.8 ± 60.0
Eicosatrienoic	C20:3 (*n-6*)	396.6 ± 35.0	422.0 ± 70.8	421.3 ± 93.6
Arachidonic	C20:4 (*n-6*)	4119.0 ± 376.6	4228.3 ± 234.0	4224.5 ± 591.4
Behenic	C22:0	600.9 ± 109.3	534.0 ± 112.8	493.6 ± 68.5
Erucic	C22:1 (*n-9*)	182.5 ± 11.8	167.9 ± 21.4	238.6 ± 109.4
Nervonic	C24:1 (*n-9*)	2583.2 ± 362.1	2581.6 ± 129.0	2578.5 ± 416.4
Length of FAs				
MCFAs	C6-12	515.8 ± 77.2	514.1 ± 69.3	497.5 ± 56.3
LCFAs	C13-21	31,102.3 ± 2368.6	31,041.9 ± 2624.9	30,955.5 ± 4997.8
VLCFAs	C22-24	3205.6 ± 391.5	2982.6 ± 373.4	2926.3 ± 379.2
Saturation of FAs				
Saturated		17,860.2 ± 1619.5	17,180.1 ± 1555.1	17,807.1 ± 2266.9
Unsaturated		16,963.5 ± 1182.3	17,358.5 ± 1229.7	16,572.3 ± 3217.1
MUFAs		11,450.2 ± 895.8	11,661.8 ± 1004.3	10,898.6 ± 2934.4
PUFAs		5513.3 ± 357.0	5696.7 ± 292.5	5673.7 ± 575.2
Ratio Sat/Unsat		1.1 ± 0.1	1.0 ± 0.0	1.1 ± 0.2

## Data Availability

Datasets cannot be publicly shared due to privacy issues. They can be provided by email upon request.
